# Non-criteria manifestations in primary antiphospholipid syndrome: a French multicenter retrospective cohort study

**DOI:** 10.1186/s13075-022-02726-9

**Published:** 2022-01-25

**Authors:** Alexis F. Guédon, Jennifer Catano, Laure Ricard, Charlotte Laurent, Claire de Moreuil, Geoffrey Urbanski, Sophie Deriaz, Grigorios Gerotziafas, Ismail Elalamy, Alexandra Audemard, Francois Chasset, Sonia Alamowitch, Jérémie Sellam, François Maillot, Jean Jacques Boffa, Ariel Cohen, Noémie Abisror, Olivier Fain, Arsène Mekinian

**Affiliations:** 1 AP-HP, Hôpital Saint-Antoine, Service de Médecine Interne and Inflammation-Immunopathology-Biotherapy Department (DMU 3iD), Sorbonne Université, F-75012 Paris, France; 2 Service de Médecine Interne, CHRU de Brest, Brest, France; 3 Service de Médecine Interne et Immunologie Clinique, Centre Hospitalier Universitaire d’Angers, Angers, France; 4 Service de Médecine Interne, Hôpital Tours, Tours, France; 5 Sorbonne Université, AP-HP, Hôpital Tenon, Service de Hémostase et Hématologie biologique, F-75012 Paris, France; 6 Sorbonne Université, AP-HP, Hôpital Tenon, Service de dermatologie et vénérologie, F-75012 Paris, France; 7 AP-HP, Service des Urgences cérébro-vasculaires, Hôpital Pitié-Salpétrière, Centre de recherche de Saint Antoine, INSERM, UMRS 938, Sorbonne Université, Paris, France; 8 Sorbonne Université, AP-HP, Hôpital Saint-Antoine, Service de rhumatologie, F-75012 Paris, France; 9 Sorbonne Université, AP-HP, Hôpital Tenon, Service de népjrologie, F-75012 Paris, France; 10 Sorbonne Université, AP-HP, Hôpital Saint-Antoine, Service de cardiologie, F-75012 Paris, France

**Keywords:** Antiphospholipid antibodies, Antiphospholipid syndrome, Non-criteria antiphospholipid syndrome

## Abstract

**Background:**

From this retrospective study, we aimed to (1) describe the prevalence and characteristics of non-criteria features in primary antiphospholipid syndrome (p-APS) and (2) determine their prognostic value.

**Methods:**

This retrospective French multicenter cohort study included all patients diagnosed with p-APS (Sydney criteria) between January 2012 and January 2019. We used Kaplan-Meier and adjusted Cox proportional hazards models to compare the incidence of relapse in p-APS with and without non-criteria manifestations.

**Results:**

One hundred and seventy-nine patients with p-APS were included during the study time, with a median age of 52.50 years [39.0; 65.25] and mainly women (*n* = 112; 62.6%). Among them, forty-three patients (24.0%) presented at least one non-criteria manifestation during the follow-up: autoimmune cytopenias (*n* = 17; 39.5%), Libman Sachs endocarditis (*n* = 5; 11.6%), APS nephropathy (*n* = 4; 9.3%), livedo reticularis (*n* = 8; 18.6%), and neurological manifestations (*n* = 12; 27.9%). In comparison to p-APS without any non-criteria manifestations (*n* = 136), p-APS with non-criteria features had more arterial thrombosis (*n* = 24; 55.8% vs *n* = 48; 35.3%; *p* = 0.027) and more frequent pre-eclampsia (*n* = 6; 14.3% vs *n* = 4; 3.1%; *p* = 0.02). The prevalence of triple positivity was significantly increased in patients with non-criteria features (*n* = 20; 47.6% vs *n* = 25; 19.8%; *p* = 0.001). Patients with p-APS and non-criteria manifestations (*n* = 43) received significantly more additional therapies combined with vitamin K antagonists and/or antiaggregants. Catastrophic APS (CAPS) tended to be more frequent in p-APS with non-criteria features (*n* = 2; 5.1% vs none; *p* = 0.074).

The p-APS with non-criteria manifestations had significantly increased rates of relapse (*n* = 20; 58.8% vs 33; 33.7%; *p* = 0.018) in bivariate analysis, but in survival analyses, the hazard ratio (HR) of relapse was not significantly different between the two groups (HR at 1.34 [0.67; 2.68]; *p* = 0.40).

**Conclusions:**

The presence of non-criteria features is important to consider, as they are associated with particular clinical and laboratory profiles, increased risk of relapse, and need for additional therapies. Prospective studies are necessary to better stratify the prognosis and the management of p-APS.

## Background

Antiphospholipid syndrome (APS) is a systemic autoimmune disease characterized by vascular thrombosis, pregnancy morbidity, and persistent antiphospholipid antibodies (APL). The classification Sydney criteria consider the arterial and venous thromboses, with or without adverse obstetrical features of APS [1]. Several other features, called non-criteria manifestations, can be associated with thrombotic and obstetrical APS features [2]. These non-criteria manifestations include immune thrombocytopenia and autoimmune hemolytic anemia, livedo reticularis, Libman Sachs endocarditis, APS nephropathy, and neurological manifestations such as migraine, chorea, and longitudinal myelitis. Although these non-criteria manifestations are not specific to primary APS, some studies suggest that their presence could be associated with an increased risk of thrombosis and could be thus defined as a “high risk” APS subtype [3, 4]. Large data about primary APS (p-APS) with non-criteria manifestations and their prognostic value remain understudied. Studies about the prevalence of these various “non”-criteria” APS in p-APS and their various management remain to be better described.

From this retrospective study, we aimed to (1) describe the prevalence and characteristics of non-criteria features in a multicenter cohort of patients with p-APS and (2) determine their prognostic value in comparison to p-APS without any non-criteria features regarding overall and relapse-free survivals.

## Methods

### Study design

All patients diagnosed with a p-APS (Sydney criteria) between January 2012 and January 2019 from departments of internal medicine, rheumatology, nephrology, neurology, dermatology, cardiology, and hematology of Saint Antoine and Tenon hospitals from Paris and university hospitals of Brest and Tours were included in this retrospective French multicenter cohort study. Patients with systemic lupus erythematosus (SLE) or other systemic autoimmune diseases were excluded. All data, including clinical, laboratory, and treatment variables, were collected by a clinician from the medical records during the first in-hospital contact and considered as baseline parameters. The presence of non-criteria manifestations was recorded as follows: immune thrombocytopenia and/or autoimmune hemolytic anemia, livedo reticularis, Libman Sachs endocarditis, APS nephropathy, and neurological disorders among multiple sclerosis-like disease, chorea, and seizure. Migraine was considered as a non-criteria manifestation if associated with another non-criteria feature and/or abnormal magnetic resonance imaging. These features were extracted from various centers’ data in a homogeneous standardized file by LR and CL and checked by AM. Combined APS patients include patients with both thrombotic APS phenotype and obstetrical APS phenotype. An ethical committee was not required for this observational study according to Helsinki law and the French institutional committee.

### Statistical analysis

Descriptive analyses were expressed as proportions (%) for categorical variables and medians with ranges for continuous variables. First, we compared phenotypes from all p-APS patients with and without non-criteria manifestations, using the non-parametric Fisher test (for qualitative variables) and the non-parametric Wilcoxon test (for quantitative variables). We used Kaplan-Meier and adjusted Cox proportional hazards models to compare the incidence of relapse in p-APS with and without non-criteria manifestations. Sex, vitamin K antagonists, and triple APL positivity status were considered as potential confounders according to the literature [5, 6]. Proportional hazards assumptions were tested based on analysis of Schoenfeld residuals and no interaction was found between variables. Data were imputed for missing data using a multiple imputation technique. A two-sided *p* value < 0.05 was considered as significant. *p* values have not been adjusted for multiple testing and should not allow inference interpretation. All analyses were performed using R software 3.6.0 version for Mac (Foundation for Statistical Computing, Vienna, Austria).

## Results

### Prevalence of non-criteria manifestations

One hundred and seventy-nine patients with p-APS were included during the study time, with a median age of 52.50 years [39.0; 65.25] and mainly women (*n* = 112, 62.6%). Among them, forty-three patients (24.0%) presented at least one non-criteria manifestation during the follow-up (Table 1). These non-criteria manifestations were autoimmune cytopenias (*n* =17; 39.5%) (immune thrombocytopenia in 13 cases, Evan’s syndrome in three cases, and autoimmune hemolytic anemia in one case), Libman Sachs endocarditis (*n* = 5; 11.6%), APS nephropathy (*n* = 4; 9.3%), livedo reticularis (*n* = 8; 18.6%), and neurological manifestations (*n* = 12; 27.9%). Thrombotic APS was the most frequent type of APS associated with non-criteria features (*n* = 26; 60.5%), and combined APS was the most frequent APS phenotype in association with Libman Sachs endocarditis (*n* = 3; 60%).

**Table 1:** Non-criteria manifestations among primary APS patients

Total number = 43	Autoimmune cytopenia	APS nephropathy	Libman-Sachs endocarditis	Neurological non-criteria	Livedo reticularis
Number	17 (39.5)	4 (9.3)	5 (11.6)	12 (27.9)	8 (18.6)
Type, *n*	ITP = 13 AIHA = 1 Evan’s syndrome = 3	-	-	Multiple sclerosis-like disease = 4 Migraine = 6 Lymphocytic recurrent meningitides = 1 Seizures = 1	-
Associated non-criteria manifestations	APS nephropathy Livedo reticularis	ITP Livedo reticularis	Migraine Livedo reticularis	Libman-Sachs endocarditis	ITP APS nephropathy Libman-Sachs endocarditis
Thrombotic phenotype (pure), *n* (%)	10 (58.8)	2 (50.0)	2 (40.0)	9 (75.0)	4 (50.0)
Obstetrical phenotype (pure), *n* (%)	3 (17.6)	0 ( 0.0)	0 (0.0)	3 (25.0)	0 (0.0)
Combined APS, *n* (%)	4 (23.5)	2 ( 50.0)	3 (60.0)	0 (0.0)	4 (50.0)
Triple positivity, *n* (%)	10 (58.8)	4 (100.0)	3 (60.0)	3 (25.0)	4 (57.1)
Relapse, *n*/total *n* (%)	10/12 (83.3)	4/4 (100.0)	1/5 (20.0)	3/10 (30.0)	4/5 (80.0)

*AIHA* autoimmune hemolytic anemia, *APS* antiphospholipid syndrome, *ITP* immune thrombocytopenic purpura

### Biological and clinical profiles of non-criteria p-APS

In comparison to p-APS without any non-criteria manifestations (*n* = 136), p-APS with non-criteria features had more arterial thrombosis (*n* = 24; 55.8% vs *n* = 48; 35.3%; *p* = 0.027) and more frequent pre-eclampsia (*n* =6; 14.3% vs *n* = 4; 3.1%; *p* = 0.02) (Table 2). Whereas the frequencies of various APL were similar between p-APS with and without non-criteria manifestations, the prevalence of triple positivity was significantly increased in patients with non-criteria features (*n* = 20; 47.6% vs *n* = 25; 19.8%; *p* = 0.001).

**Table 2:** APS characteristics and outcomes in patients with and without non-criteria manifestations

	Primary APS with non-criteria manifestations (***n*** = 43)	Primary APS without non-criteria manifestations (***n*** = 136)	***p*** value
Male sex, *n* (%)	14 (32.6)	53 (39.0)	0.564
Age, years, median [IQR]	53.00 [38.50, 69.50]	52.00 [39.00, 65.00]	0.758
**APS features**			
Thrombotic phenotype (pure), *n* (%)	26 (60.5)	86 (63.2)	0.884
Obstetrical phenotype (pure), *n* (%)	6 (14.0)	22 (16.2)	0.913
Combined APS, *n* (%)	11 ( 25.6)	29 ( 21.3)	0.708
Number of thrombosis, *n* (%)			0.856
None	7 (16.3)	24 (17.6)	
One	27 (62.8)	79 (58.1)	
Two or more	9 (20.9)	33 (24.3)	
Arterial thrombosis, *n* (%)	24 (55.8)	48 (35.3)	0.027
Venous thrombosis, *n* (%)	17 (39.5)	73 (53.7)	0.149
Miscarriages, *n* (%)	6 (14.3)	13 (9.9)	0.615
Intrauterine deaths, *n* (%)	6 (14.3)	22 (16.8)	0.886
Prematurity, *n* (%)	3 (7.1)	7 (5.3)	0.956
IUGR, *n* (%)	3 (7.1)	7 (5.4)	0.965
Pre-eclampsia, HELLP syndrome, *n* (%)	6 (14.3)	4 (3.1)	0.020
CAPS, *n* (%)	2 (5.1)	0 (0.0)	0.074
**Cardiovascular risk factors**			
Arterial hypertension, *n* (%)	14 (51.9)	38 (38.4)	0.299
Dyslipidemia, *n* (%)	8 (29.6)	23 (23.2)	0.666
Tobacco, *n* (%) = 1 (%)	7 (35.0)	13 (21.3)	0.351
Diabetes mellitus, *n* (%)	4 (19.0)	8 (12.5)	0.699
Overweight, *n* (%)	5 (21.7)	25 (31.6)	0.511
**Laboratory data**			
Anti-cardiolipid IgG, IU, median [IQR]	22.40 [5.00, 57.00]	18.00 [4.90, 63.00]	0.868
Anti-cardiolipid IgG positive, *n* (%)	24 (57.1)	57 (49.6)	0.509
Anti-cardiolipid IgM, IU, median [IQR]	11.00 [2.00, 53.35]	10.00 [2.20, 38.20]	0.980
Anti-cardiolipid IgM positive, *n* (%)	17 (43.6)	46 (39.7)	0.807
Anti-β2Gp1 IgG, IU, median [IQR]	17.10 [2.00, 60.00]	4.00 [1.00, 25.00]	0.312
Anti-β2Gp1 IgG positive, *n* (%)	18 (42.9)	39 (33.6)	0.379
Anti-β2Gp1 IgM, IU, median [IQR]	3.00 [1.00, 19.30]	3.00 [1.00, 29.45]	0.930
Anti-β2Gp1 IgM positive, *n* (%)	13 (32.5)	36 (31.0)	1.000
LAC, *n* (%)	19 (63.3)	51 (61.4)	1.000
Triple positivity, *n* (%)	20 (47.6)	25 (19.8)	0.001
**Treatment and outcomes**			
Antinuclear antibodies, *n* (%)	15 (40.5)	22 (23.2)	0.075
Vitamin K antagonists, *n* (%)	31 (77.5)	81 (64.8)	0.193
Antiplatelet therapy, *n* (%)	19 (50.0)	58 (45.3)	0.746
Hydroxychloroquine, *n* (%)	12 (31.6)	19 (14.7)	0.035
Steroids, *n* (%)	12 (34.3)	18 (14.4)	0.016
Relapse, *n*/total *n* (%)	20/34 (58.8)	33/98 (33.7)	0.018
Death, *n*/total *n* (%)	5/37 (13.5)	5/103 (4.9)	0.167
Time to relapse, years, median [IQR]	3.58 [1.23, 12.54]	1.71 [0.48, 5.77]	0.260
Follow-up, years, median [IQR]	5.37 [0.96, 11.98]	2.95 [1.09, 7.83]	0.191

*APS* antiphospholipid syndrome, *CAPS* catastrophic antiphospholipid syndrome, *HELLP* hemolysis, elevated liver enzymes, and low platelet count, *IUGR* intrauterine growth restriction, *LAC* lupus anticoagulant

Triple-positive p-APS with non-criteria manifestations (*n* = 20) had significantly increased rates of relapses (12 (57%) vs 6 (31%); *p* = 0.03) in comparison to triple-positive APS without non-criteria features (*n* = 25), whereas other characteristics (age, follow-up, type of APS, use of immunosuppressive drugs, and hydroxychloroquine) were not significantly different.

### Outcome and management of p-APS with non-criteria manifestations

Patients with p-APS and non-criteria manifestations (*n* = 43) received significantly more additional therapies combined with vitamin K antagonists and/or antiaggregants (Table 2). These additional therapies were mainly hydroxychloroquine (*n* = 12; 31.6% vs *n* = 19; 14.7%; *p* = 0.035) and steroids (*n* = 12; 34.3% vs *n* = 18; 14.4%; *p* = 0.016). During the median follow-up of 5.37 years in p-APS with non-criteria manifestations and 2.95 years in those without any non-criteria features (*p* = 0.19), the death rates were not significantly different between the two groups (*n* = 5; 13.5% vs *n* =5; 4.9%; *p* = 0.17). While rare, catastrophic APS (CAPS) tended to be a more frequent complication of p-APS with non-criteria features (*n* = 2; 5.1% vs none; *p* = 0.074).

### Factors associated with relapse

**Fig. 1:**
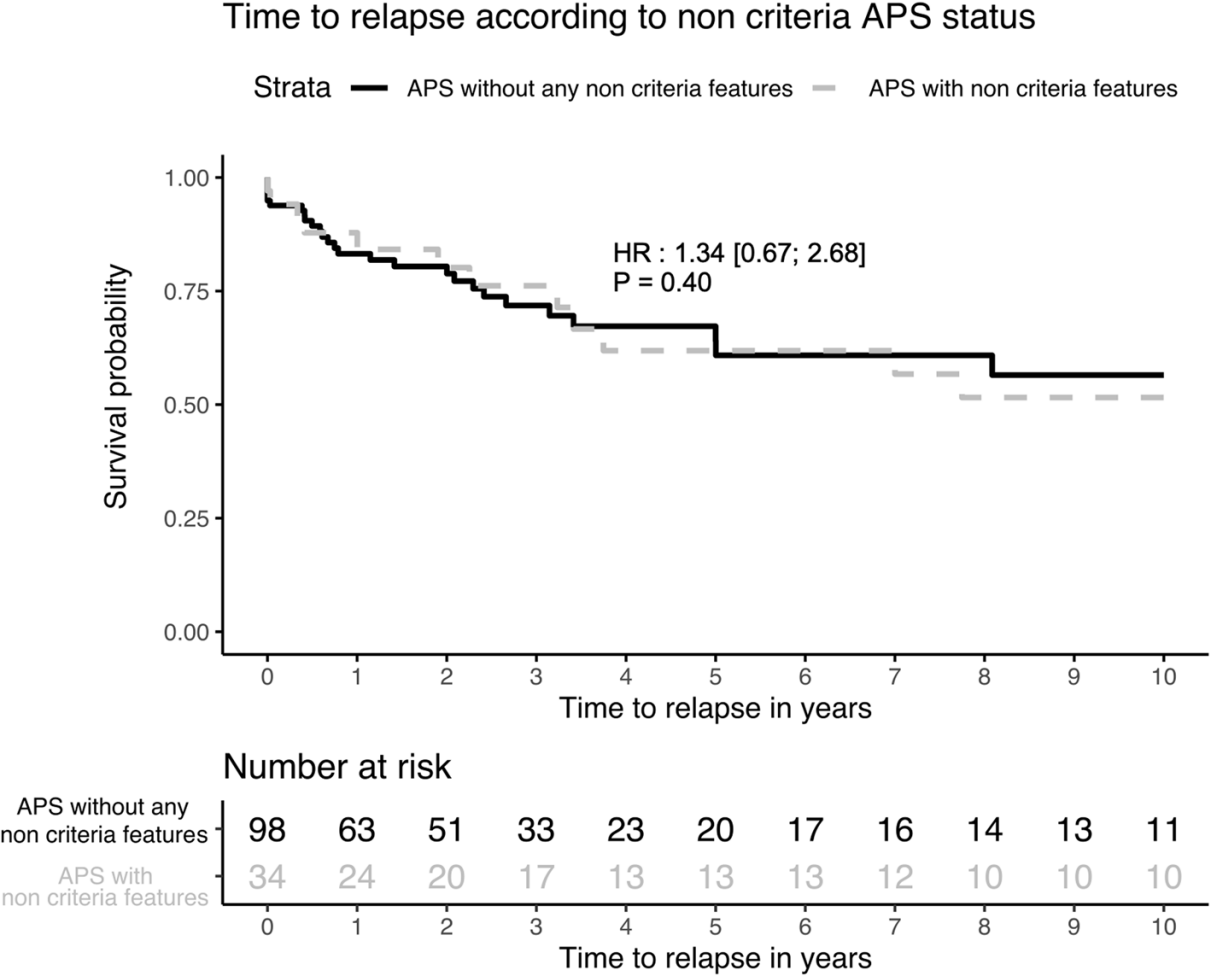
Time to relapse according to non-criteria APS status

The p-APS with non-criteria manifestations had significantly increased rates of relapse (*n* = 20; 58.8% vs 33; 33.7%; *p* = 0.018) in bivariate analysis, but in survival analyses, the hazard ratio (HR) of relapse was not significantly different between the two groups (HR at 1.34 [0.67; 2.68]; *p* = 0.40) (Fig. 1). Bivariate analysis of factors associated with relapse showed that relapsing patients had significantly more combined APS profile (*n* = 17; 32.1% vs *n* = 4; 5.1%; *p* < 0.001), a previous history of pre-eclampsia (*n* = 7; 13.7% versus *n* = 2; 2.6%; *p* = 0.042), and more non-criteria features (*n* = 20; 37.7% vs *n* = 14; 17.7%; *p* = 0.018) (Table 3). In multivariate analysis, none of these risk factors was independently associated with the risk of relapse (Table 4).

**Table 3:** Factors associated with relapse: comparison of patients with and without relapses

	APS patients without any relapse during follow-up (***n*** = 79)	APS patient with relapse during follow-up (***n*** = 53)	***p*** value
Male sex, *n* (%)	23 (29.1)	21 (39.6)	0.286
Age, years, median [IQR]	53.50 [38.75, 66.25]	60.50 [40.75, 69.25]	0.343
**APS features**			
Thrombotic phenotype (pure), *n* (%)	61 (77.2)	33 (62.3)	0.096
Obstetrical phenotype (pure), *n* (%)	14 (17.7)	4 (7.5)	0.158
Combined APS, *n* (%)	4 (5.1)	17 (32.1)	<0.001
Number of thrombosis, *n* (%)			<0.001
None	15 (19.0)	6 (11.3)	
One	53 (67.1)	22 (41.5)	
Two or more	11 (13.9)	25 (47.2)	
Arterial thrombosis, *n* (%)	28 (35.4)	23 (43.4)	0.461
Venous thrombosis, *n* (%)	40 (50.6)	32 (60.4)	0.356
Miscarriages, *n* (%)	10 (13.2)	6 (11.8)	1.000
Intrauterine deaths, *n* (%)	5 (6.6)	5 (9.8)	0.745
Prematurity, *n* (%)	4 (5.3)	4 (7.8)	0.830
IUGR, *n* (%)	1 (1.3)	3 (5.9)	0.362
Pre-eclampsia, HELLP syndrome, *n* (%)	2 (2.6)	7 (13.7)	0.042
CAPS, *n* (%)	0 (0.0)	2 (3.8)	0.304
**Cardiovascular risk factors**			
Arterial hypertension, *n* (%)	23 (39.0)	13 (41.9)	0.964
Dyslipidemia, *n* (%)	11 (18.6)	8 (25.8)	0.603
Tobacco, *n* (%) = 1 (%)	9 (17.0)	7 (35.0)	0.179
Diabetes mellitus, *n* (%)	9 (15.8)	3 (15.0)	1.000
Overweight, *n* (%)	7 (15.9)	9 (32.1)	0.185
**Non-criteria features**			
Non-criteria features, *n* (%)	14 (17.7)	20 (37.7)	0.018
Number of non-criteria features, median [IQR]	0.00 [0.00, 0.00]	0.00 [0.00, 1.00]	0.007
**Laboratory data**			
Triple positivity, *n* (%)	17 (23.0)	18 (36.7)	0.147
Antinuclear antibodies, *n* (%)	11 (19.6)	11 (29.7)	0.384
**Treatment and outcomes**			
Vitamin K antagonists, *n* (%)	39 (52.7)	45 (90.0)	<0.001
Antiplatelet therapy, *n* (%)	30 (39.0)	26 (53.1)	0.171
Hydroxychloroquine, *n* (%)	6 (7.8)	16 (32.7)	0.001
Steroids, *n* (%)	10 (13.0)	14 (29.8)	0.039
Death, *n* (%)	5 (13.5)	5 (4.9)	0.167
Follow-up, years, median [IQR]	5.37 [0.96, 11.98]	2.95 [1.09, 7.83]	0.191

*APS* antiphospholipid syndrome, *CAPS* catastrophic antiphospholipid syndrome, *HELLP* hemolysis, elevated liver enzymes, and low platelet count, *IUGR* intrauterine growth restriction

**Table 4:** Univariate and multivariate factors associated with relapse

	HR	95% ***CI*** (HR)	***p*** value
Univariable Cox model (outcome:relapse)			
APS non-criteria features	1.34	[0.67; 2.68]	0.402
Multivariable Cox model (outcome:relapse)			
APS non-criteria features	1.35141	[0.63623; 2.87052]	0.43334
Male sex	1.39057	[0.7032; 2.74984]	0.34323
Vitamin K antagonists	2.45312	[0.89569; 6.71861]	0.08081
Triple positivity	0.80880	[0.3626; 1.80409]	0.60416

*APS* antiphospholipid, *CI* confidence interval, *HR* hazard ratio

## Discussion

From this cohort of p-APS, the main findings are that (1) p-APS with non-criteria features have an increased prevalence of severe features such as arterial thrombosis and pre-eclampsia, (2) triple positivity is increased in p-APS with non-criteria features, and (3) p-APS with non-criteria features might have a poorer prognosis, as suggested by the increased need for additional therapies.

There is still no clear consensus on the exact definitions of non-criteria APS. A recent consensus paper proposed a classification in four categories, including “clinical non-criteria APS patients,” who were patients presenting non-criteria manifestations and APL positivity fulfilling the classification criteria [7]. The prevalence of non-criteria features in p-APS varies according to the studied cohorts and depends on the inclusion criteria, in particular, the exclusion of associated SLE. In an Italian study on 200 women with p-APS ongoing a pregnancy, 39 (19.5%) had non-criteria manifestations, mainly livedo reticularis, valvulopathy, and autoimmune cytopenias [8]. Among 99 female obstetrical APS patients from the APS ACTION registry, livedo reticularis was present in 35%, thrombocytopenia in 44%, and valvulopathy in 15%, but the presence of non-criteria features was not associated with the first thrombosis [9]. In the European registry of 1000 p-APS and SLE-associated APS, non-criteria features were commonly observed, including thrombocytopenia (8.7%), livedo reticularis (8.1%), autoimmune hemolytic anemia (4%), valve thickening/dysfunction (4.6%), and epilepsy (3.2%) [10]. The prevalence of these non-criteria features in cohorts of p-APS is still not well-established, and a third of our patients have at least one non-criteria feature in this unselected p-APS cohort without any SLE.

Triple positivity was recently demonstrated as a particular laboratory feature associated with an increased risk of thrombosis and obstetrical relapses and a severe APS course. Patients with APS and triple positivity for aPL are at high risk of developing future thromboembolic events with a cumulative incidence of thrombosis at 12.2% (95% *CI*, 9.6–14.8) after 1 year, 26.1% (95% *CI*, 22.3–29.9) after 5 years, and 44.2% (95% *CI*, 38.6–49.8) after 10 years [5]. Among APL asymptomatic carriers, none of the baseline characteristics was predictive of risk of first thrombosis, and the strongest association was found in triple aPL-positive carriers: odds ratio 3.38 (95% *CI* 1.24–9.22) [11]. Patients with triple aPL positivity had a higher rate of pregnancy complications, despite the fact that they were more frequently receiving low-dose aspirin with low molecular weight heparin [12]. The increased prevalence of triple-positive APS was also noted near 50% of refractory APS patients from the European retrospective cohort [13]. In our study, near half of APS with non-criteria features presented a triple positivity (versus 20% in those without non-criteria features), conferring risk of severe course and risk of relapse. However, one major limitation of our study was the small size of our sample with available follow-up date, resulting in low statistical power. This might explain the reason why we do not find any difference between patients with and without non-criteria manifestation in our survival analyses, though the bivariate analysis was significantly different. The not-standardized definition of non-criteria APS features could be another important publication bias.

The definition and stratification of risk profile in p-APS are of particular interest, as the management of APS is still mainly based on obstetrical or thrombotic clinical phenotype. Indeed, despite several data about the unfavorable outcome, in particular of triple-positive patients, of p-APS patients with positive antinuclear autoantibodies and lupus-like profile (unpublished personal data) or increased Global Anti-Phospholipid Syndrome Score (GAPPS) score, there is actually no real therapeutic adjustments according to these various prognostic risk factors. The value of additional therapies, in particular in obstetrical APS, has been studied, showing promising results using low-dose steroids, hydroxychloroquine, or plasma exchanges [14, 15]. The value of additional therapies, particularly hydroxychloroquine, as illustrated in our cohort, should be better determined, in the specific subset of patients with non-criteria features [16, 17].

## Conclusion

The presence of non-criteria features in p-APS patients is important to consider, as they are associated with particular clinical and laboratory profiles, increased risk of relapse, and need for additional therapies. Prospective studies are necessary to better stratify the prognosis and management of p-APS.

## Data Availability

Yes. Arsene Mekinian consented to the full data availability.

## Abbreviations

AIHA: Autoimmune hemolytic anemia
APL: Antiphospholipid
APS: Antiphospholipid syndrome
CAPS: Catastrophic antiphospholipid syndrome
GAPPS: Global Anti-Phospholipid Syndrome Score
HELLP: Hemolysis, elevated liver enzymes, and low platelet count
ITP: Immune thrombocytopenic purpura
IUGR: Intrauterine growth restriction
LAC: Lupus anticoagulant
p-APS: Primary antiphospholipid syndrome
SLE: Systemic lupus erythematosus
